# Interactions between Mycorrhizal Fungi, Tea Wastes, and Algal Biomass Affecting the Microbial Community, Soil Structure, and Alleviating of Salinity Stress in Corn Yield (*Zea* *mays* L.)

**DOI:** 10.3390/plants7030063

**Published:** 2018-08-08

**Authors:** Salwan Al-Maliki, Mugtaba AL-Masoudi

**Affiliations:** Soil and Water Science Department, College of Agriculture, Al-Qasim Green University, Al Qasim 13239, Iraq; mors14900@gmail.com

**Keywords:** mycorrhizal fungi, soil respiration, mean weight diameter (MWD), total bacteria and fungi, corn plant, tea wastes, algal biomass, saline soil

## Abstract

Soil salinity has an adverse impact on soil biological properties and growth of corn plant, majorly in arid and semi-arid lands. A mesocosm experiment was conducted to investigate the effect of mycorrhizal fungi (M) (*Glomus mosseae*), tea wastes (T), algal dried biomass (A), and their combinations on soil respiration, total bacteria, total fungi, soil mean weight diameter (MWD), and corn yield (*Zea* *mays* L.)*.* under saline and non-saline soils. Results showed that M, T, and A treatments increased significantly CO_2_ release compared to the control. Whereas, M significantly decreased CO_2_ release compared to T and A treatments. In non-saline soil, M increased greatly MWD, bacterial and fungal counts, and infection rate. Whereas, the opposite was true in the saline soil; neither M nor T improved bacterial communities and MWD. However, in the saline soil, M + T was highly efficient in improving MWD, SOC, bacterial and fungal counts, infection rate, and corn grain yield. It can be suggested that the inoculation of mycorrhizal fungi with tea wastes in saline soils considered an important strategy that increases the toleration of the corn plant to salinity by improving soil microbial activity, MWD, SOC, infection rate, and total grain yield.

## 1. Introduction

In recent years, soil salinity has become one of the most severe ecological factors in many countries of the world particularly in the arid and semi-arid lands [1,2]. It is reported that crop yields have increasingly decreased in saline soil due to the exposure of the plant to the extra osmotic pressure, ion toxicity is caused by the high concentrations of Na^+^ and Cl^−^ [3,4,5]. The negative intensified effects of salinity in the arid and semi-arid lands were due to the heavy fertilization and improper irrigation [6]. One of the vital ways to increase the plant toleration to salinity stresses is to incorporate the *Arbuscular mycorrhizal* fungi (AM fungi) in soils [7]. AM fungi are capable of increasing the endurance of plants to salt stress by enhancing plant nutrient uptake and ion balance [8,9]. Moreover, AM fungi alleviates salt stress in plant and have a capacity to protect soil enzymes and soil organic matter [10,11], and concurrently facilitating the uptake of water [12].

The plant litter decomposition in soil is a key driver of ecosystem processes and can be an important contributor to soil carbon (C) storage. They are several biotic and abiotic factors such as temperature, soil moisture, and soil organisms governing the rate of litter decomposition. AM fungi are a prime group of organisms associated with litter decomposition. A previous study [13] found that AM fungi reduced the decomposition rates of woody material. Less decomposition rate in the presence of AM can lead to stabilization of C within soil aggregates, contributing to soil C storage into the soil [14,15]. Similarly, authors of a past paper [16] noted that AM fungi reduced CO_2_ release from the soil, which led to the sequestration of carbon in the semiarid lands. However, the effect of AM fungi on the decomposition rate of litter is still not consistent. Authors of a past paper [17] observed that the presence of AM fungi caused a rapid decomposition of plant litter, especially when conditions of elevated CO_2_ and nitrogen (N) concentrations are applied. Likewise, authors of a past paper [18] noticed a reduction of C in the soil when AM fungi were present and concluded that AM fungi promoted litter decomposition by stimulating the activity of bacteria. Moreover, mycorrhizal fungal species have an ability to produce pectinases, cellulases, and hemicellulases which are responsible for the decomposition process [19]. However, the degradation of organic matter by AM fungi has not been considered direct [20] because AM fungi have not acquired saprotrophic abilities but depend on the saprotrophic microbes to decompose recalcitrant organic matter resulting in more inorganic N release to be captured by AM fungi [21]. Furthermore, AM fungi can indirectly affect the decomposition process by producing enormous exudates which play a vital role in encouraging microbial community in soil to decompose the organic materials. For instance, authors of a past paper [22] reported that AM fungal hyphal exudates of low molecular weight sugars and organic acids increased the soil bacterial community. While, authors of a past paper [23] found that AM fungi hyphal exudates promoted some soil microbes but inhibited other microorganisms. Similarly, authors of a past paper [24] outlined that soil microbes were suppressed by AM fungi in the presence of cellulose, although, this study examined this relationship on the artificial media. It seems therefore that the types of organic substrates have influenced the relationship between AM fungi and microbial community [25]. However, the authors of a past paper [26] reviewed the relationship between AM fungi and soil microorganisms and reported that AM fungi increased, decreased, or have no influence on the soil microorganisms. Consequently, little is known about the effect of AM fungi on the soil microbial community.

Tea wastes play a fundamental role in agriculture in many regions. It has been reported that tea waste increased soil aggregate stability of the degraded lands [27]. Moreover, they have an ability to manipulate the saline soil by decreasing the electric conductivity of the soil and increasing the productivity of the plant [28]. Furthermore, the compositions of tea wastes contain phenols and amino acids which might modify the higher pH of saline soil, leading to an improved soil microbial activity. Algae, on another hand, have a great efficiency in improving soil properties and the plant growth. Authors of a past paper [29] showed that mineralization of soil organic carbon increased when algal dried biomass mixed with the soil. These results confirmed that the algae accelerated soil organic matter decomposition and increased the available nutrients in the soil. Furthermore, algal biomass has carbohydrates, plant growth regulators like cytokines, and gibberellins which consider an effective contributor to the plant growth [30].

No studies have investigated the interactions between AM fungi, tea waste and algal dried biomass under saline and non-saline soils. Furthermore, these studies have mostly been conducted in the artificial media or sterilized soils, neglecting the natural habitat of the field soils. Consequently, it is unknown whether the interactions between AM fungi, tea waste, and algae dried biomass are beneficial to AM fungi and corn plant to overcome the salinity stress and increase microbial activity, microbial community, soil aggregation, infection rate, and growth of the corn plant. The proposed hypothesis was that the combination of AM fungi with tea waste or algal dried biomass in saline and non-saline soils can revive the microbial community, infection rate, and increase the soil aggregate formation and growth of the corn plant. Therefore, the objective of this study is to evaluate the effect of AM fungi, tea waste, and algal dried biomass on soil microbial activity, microbial community, mean weight diameter, infection rate, and growth of corn plant under saline and non-saline soils. 

## 2. Materials and Methods

### 2.1. Experimental Site 

Soil samples were taken from two fields to a depth of 0–30 cm. Both fields were located in the Abi Gharaq area (32°30′02.8″ N, 44°19′49.7″ E) in the Babylon Governorate. One of the fields was suffered from the salinity impact (Ec 7.9 dSm^−1^) while the other field was non-saline (Ec 3.6 dSm^−1^) and it was frequently cultivated in wheat plants. These zones are categorized based on a previous paper [31], as Arid–Desert-hot zones (BWh). The principal features of the Babylon climate are high temperature, low rainfall and low humidity, giving an average annual temperature of 30.1 °C, an average annual rainfall of 101 mm, and low humidity 35%. The soil was dried aerobically and passed through a 4 mm diameter sieve. Pots of a 50 kg weight and a 50 cm depth were filled equally and placed in the greenhouse at the College of Agriculture, AL-Qasim Green University. The chemical and physical properties of the soils were measured as described previously [32] and are presented in Table 1. Eight treatments (control, algae, tea waste, mycrorrhiza, mycorrhiza + algae, mycorrhiza + tea waste, algae + tea waste, and mycorrhiza + algae + tea waste) were used in both experiments. The number of mesocosms for each experiment was 24 with a total of 48 mesocosms for the saline and non-saline soil. All mesocosms were randomly distributed in the greenhouse. There was a control treatment that did not receive spores. All mesocosms were sown in seeds of the maize plant (*Zea mays* L*.*) on 15 March 2017 with five hybrid seeds for each mesocosm to a depth of 3 cm. Moreover, seedlings were thinned to three seedlings per pot. Pots were weighed gravimetrically to maintain the soil moisture content close to the field capacity. The field capacity was 25% and 20% for the non-saline and the saline soil respectively. Corn plant was harvested on 15 July 2017. Soil samples were taken from the rhizosphere area (rhizosphere zone is 0–2 mm distance from roots) and analyzed to measure microbial activity, aggregate stability, the abundance of bacteria and fungi, organic carbon, infection rate, spores density, and yield production. 

### 2.2. Inoculation of Mycorrhiza Fungi

AM fungi (*Glomus mosseae*) were obtained from the Agriculture Research Office **(**AGROF). AM fungi spores were estimated by the wet sieving and decanting method as proposed by [33]. The density of spores was 42 per 1 g soil. 200 g of mycorrhizal fungal inoculums was added to the soil, half of the mycorrhizal fungal inoculums were placed at a depth of 5 cm, while the other half was mixed with the topsoil.

### 2.3. Collection of Algae

The algal samples were collected from the AL-Kefal River in the Babylon province. Algae samples were classified according to the classification key [34]. The algal species were *chlamydomonas* sp., *cocconeis* sp., *anabanena* sp*.*

The various algal species were washed by water properly to remove any weeds. Algae were then dried into the oven at 50 °C for two days. The dried algae were then grinded using an electric grinder and mixed with the soil at a depth of 30 cm. 250 g algal dried biomass per pot was applied (equivalent to 5 g/kg soil).

### 2.4. Tea Waste

The source of tea wastes was from the local university club. The black tea wastes were air-dried and grinded with an electric mill to less than 1 mm in a diameter. Tea wastes were added at a rate of 350 g per pot (equivalent to 7 g per kilo of soil) and mixed at a depth of 30 cm. The chemical composition of tea and algae are shown in Table 2. The analysis of plant tissue was measured according to a method described previously [35].

### 2.5. Chemical Analysis

#### 2.5.1. Soil Respiration

Soil respiration was measured using the alkali trap method [36]. This method is based on the measurement of CO_2_ released during the microbial activity in the soil. Twenty g of soil was placed in a sealed flask. A 10 mL beaker was situated inside the flask contains 5 mL solution of NaOH (1 M) and then incubated for 2, 4, 8, 12, and 30 days at 25 °C. At the end of each incubation period, three drops of the phenolphthalein indicator were added to the NaOH solution changed the color to pink. The NaOH solution was neutralized using 1 M HCl and the endpoint of the titration was when the pink color changed to pale. Furthermore, a solution of BaCl_2_ (2.0 mL) of a 30% (*w*/*v*) was added to the samples before titration to precipitate the CO_3_ as BaCO_3_. The amount of carbon dioxide was calculated by the following equation:

$${\mathrm{CO}}_{2}\;\left(\frac{\mathrm{mg}}{g}\right)=\frac{\left(B-V\right)N22}{w}.$$

where B = standard HCl used to titrate NaOH in the blank (mL); V = standard HCl used to titrate NaOH in the treatment (mL); N = normality of HCl (1.00 N); 22 = equivalent weight of CO_2_; and W = dry weight of soil in the chamber (g).

#### 2.5.2. Organic Carbon

The organic carbon was estimated based on a previous method [37] by taking 10 g of soil, and placed in an oven 105 °C for 24 h to estimate the moisture content of the soil. The soil was then placed in the muffle furnace for 16 h at a temperature of 400 °C to estimates the percentage of organic matter in the soil through the following equation. LOI results were divided by 1.724 to have the percentages of soil organic carbon.

$$\%\;\mathrm{loss}\;\mathrm{ignition}=\frac{\left(\mathrm{oven}\;\mathrm{dry}\;\mathrm{soil}\;\mathrm{weight}-\;\mathrm{ignited}\;\mathrm{soil}\;\mathrm{weight}\;\right)}{\left(\mathrm{oven}\;\mathrm{dry}\;\mathrm{soil}\;\mathrm{weight}\right)}\times 100.$$

#### 2.5.3. Bacterial and Fungal Population

The bacterial and fungal population were counted using a soil dilution plate technique [38]. A serial dilution of 1 g of soil with a sterilized water to 10^−1^–10^−7^ was utilized. One mL aliquots were transferred to 20 mL of nutrient agar mediums. The growth of bacterial colonies was followed up in the Tryptic-soya agar while the growth of fungal colonies was pursued in the Martin’s Rose Bengal agar. The bacterial and fungal community were enumerated by counting colony-forming units (CFU g^−1^ of dry soil). Bacterial and fungal plates were incubated at 30 °C for 3 and 7 days, respectively. The estimation of the total bacteria and fungi per gram of diluted sample was as below:

Viable cells/g dry soil = (Mean plate count × Dilution factor)/Dry weight of soil (g)

#### 2.5.4. Mean Weight Diameter

The mean weight diameter was estimated using the wet sieving method adapted from [39]. An air-dried soil was sieved using 4 mm and 2 mm sieves to obtain 2–4 mm size fractions. A number of sieves of 2000, 1000, 500, 250, and 53 μm were used to separate soil aggregates. 25 g of 2–4 mm soil was placed on the top of the 2000 μm sieve and wet sieved for 3 min at 100 cycles/min. The aggregates fractions were oven dried at 105 °C for 24 h, weighed, and calculated according to the mean weight diameter equation as below:

$$MWD=\sum_{i=1}^{n}XiWi$$

where: *MWD*—the mean-weight diameter of water stable aggregates, *Xi*—mean diameter of each size fraction (mm), and *Wi*—the proportion of the total sample weight (WSA) in the corresponding size fraction. Higher MWD values imply higher aggregate stability.

#### 2.5.5. Estimation of Mycorrhizal Colonization Rate

The AM fungal colonization rate was measured according to a previously described method [40]. The corn roots were initially washed carefully under the tab water to free soil particles adhered to the roots regime. The roots were chopped into segments of 1.5 cm and then placed in a glass bottle contained 10% (*w*/*v*) potassium hydroxide (KOH) and incubated at 90 °C for 15 min. Roots were cleared using 10% (*w*/*v*) hydrogen peroxide for 1 min. The roots were then acidified in 10% hydrochloric acid (*w*/*v*) for 3 min, and stained with acid fuchsin which was added to the water bath for 10–15 min at 90 °C. The lactic acid was then added to the roots and a 4X optical microscope was used to indicate the percentage of colonization. 10 stained root segments with a length of 1 cm were placed on slides. Acid fuchsin was prepared by adding an 875 mL of acetic acid to 63 mL of the glycerol with 63 mL of distilled water. Colonization rate was measured as below:

The AM fungi colonization rate = infected root segments/total root segments × 100%

#### 2.5.6. Spores Numbers

AM fungi spores in soil were estimated based on wet sieving and decanting method of a previous paper [33]. Ten g of the rhizospheric soil was added to 100 mL of distilled water. The contents were centrifuged at 4000 cycles per minutes for 5 min and then the soil suspension was allowed to settle. Suspensions were decanted through a series of sieves with 35, 50, 100, and 175 μm. This processes allowed the full isolation of mycorrhizal fungal spores from the soil. The contents of each sieve were pooled for counting. 1 mL of the spore suspension was placed on the counting slide to estimate the number of spores in 10 g soil. 

### 2.6. Statistical Analysis

Minitab version 14 was used to analyze data. Two-way analysis of variance (ANOVA) was utilized to analyze microbial activity, including two factors (treatments and time); with eight treatments tested control, algae, tea waste, mycrorrhiza, mycorrhizea + algae, mycorrhiza + tea waste, algae + tea waste, and mycorrhiza + algae + tea waste. Time tested five incubation periods 0, 4, 8, 12, and 30 days. Results for the aggregate stability, the abundance of bacteria and fungi, organic carbon, infection rate, spores density, and yield production were analyzed by one-way ANOVA. Data were examined for normality using normal scores plots, and that data conformed to the assumption of normality. Further evaluations of the Means were achieved where significant treatment effects were observed using Tukey’s significance difference (HSD) test with a significance level of *p* < 0.05.

## 3. Result and Discussion

### 3.1. Effect of Treatments and Incubation Periods on Soil Respiration 

Soil respiration differed significantly after addition of treatments in saline and non-saline soils (Table 3 and Table 4). Overall, for the saline and non-saline soil, the highest value of soil respiration (*p* < 0.01) was in T treatment compared with other treatments. This might be due to a lower C:N (19:1) in tea wastes, which might have decomposed rapidly during the incubation period leading to an increase in the CO_2_ release. Additionally, T is richer in carbohydrates and nutrients, which could stimulate soil microbial activity. Authors of a previous paper [41] recorded a higher microbial activity in the more decomposable materials which had lower C:N (11:1) and higher nitrogen contents. However, it seems that the incubation period affected soil respiration; for instance, T treatment exhibited an increase in soil respiration early incubation at day 2 and 4 only and then was not significantly different from A treatments at day 8, day 12, and day 30. The possible explanation is that both T and A have broadly similar amounts of carbon and nitrogen which can uniformly support soil microbes over time. Moreover, A + T treatment showed higher significant soil respiration at day 8 than T treatment. The obvious reason could be that the combination of A with T might provide sufficient food requirements for the biological processes over a longer period. For instance, T and A are loaded in protein and nutrients which can meet the microbial requirements over the incubation period. 

Our hypothesis is that AM fungi can increase microbial activity in saline and non-saline soil. However, in our study, this hypothesis is partially true; M with or without T increased soil respiration in saline and non-saline soil compared to the control treatment which suggested an improvement in microbial activity after the inoculation of M in the soil. Soil respiration can be affected by AM fungi due to a presence of AM fungal hyphal exudates like glomalin, amino acid, and sugar [22], which is considered as a vital stimulator for microbial activity [41]. Furthermore, T and A contain protein, phenols, and amino acids [42,43] which might contribute to the modification of pH in saline soil, causing a positive enhancement in soil microbial activity. Moreover, AM fungi play an important role in the saline soils by promoting root growth [2] which might contribute to an increase in the soil microbial activity. These results are in agreement with [17] who observed that the presence of AM fungi increased the litter decomposition rapidly causing higher CO_2_ release, although they conducted the experiment in different conditions using elevated CO_2_ and nitrogen concentrations. However, it was noted that M reduced significantly CO_2_ release when mixed with either T or A compared with the signal addition of T and A treatments. The profound illustration of this status is that based on our data, there were considerable increases in MWD in M + T and M + A treatments which reflected the developments in soil structure and carbon protection; leading to a limited CO_2_ release from the soil. Authors of a previous paper [14] found that soil structure retained organic carbon from the decomposition process. This study can suggest that AM fungi might contribute to carbon sequestration and the implication for the climate change. Soil respiration differed significantly over the incubation period; the highest value of soil respiration (*p* < 0.01) was at 2 days than 30 days. This may be due to the decline of organic carbon in soil over time, which might have used by microorganisms as energy sources to form microbial cells, leading to a decreased microbial activity [44].

### 3.2. Effect of Treatments on Mean Weight Diameter (MWD)

**Figure 1:**
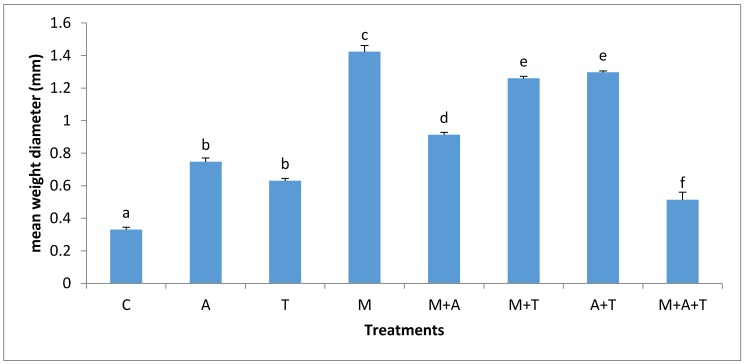
Effect of treatments control (C), algae (A), tea wastes (T), mycorrhizal fungi (M), mycorrhizal fungi + algae (M + A), mycorrhizal fungi + tea wastes (M + T), algae + tea wastes (A + T) and mycorrhizal fungi + algae + tea wastes (M + A + T), on the mean weight diameter (mm) in the non-saline soil. Bars represent standard errors. Different superscript letters represent the statistical difference (*p* < 0.05).

There were obvious differences in MWD after the application of tea wastes, mycorrhizal fungi and algal dried biomass in saline and non-saline soil. In non-saline soil, the highest significant increase of MWD (*p* < 0.01) was when M added alone compared to the combined treatments (Figure 1). There are various possible explanations for this episode; firstly, the mycorrhizal fungal myceliums have an ability to produce glomalin and soluble carbon [41] which might give important considerations to soil aggregates formation [45]. Secondly, the mycorrhizal fungal products can maximize soil microbes which have the basic role of increasing MWD. Based on our data, it was noted remarkable increases of fungal and bacterial communities when AM fungi were present in soil (Figures 3 and 4); these events might intense microbial mucilages which have a direct role in improving MWD. Furthermore, it was recognized that MWD in M + T treatment preferably increased as compared to M + A. This is because of the higher contents of polyphenol acid and carbohydrates in tea wastes which might have encouraged AM fungi and microbial community to grow substantially causing a pronounced soil aggregates formation. [46,47] concluded that soil-aggregate formation was affected largely by phenolic acids and also led to a stronger aggregates stability of soil. 

Unexpectedly, the incorporation of M with T or A significantly decreased MWD compared to the single addition of M in the non-saline soil. To the best of our knowledge, there is no study available on the combination of AM fungi with tea wastes or algal dried biomass in saline and non-saline soils. It seems that amendments of T and A had improved soil fertility due to greater amounts of polyphenol acid, amino acids, nutrients, and protein in wastes which might have reduced the dependency of the plant on mycorrhizal associations which considered very important factor controlling soil aggregate formation. In contrast, inoculation of AM fungi without organic wastes could not considerably increase the soil fertility, thereby these conditions might force the plant to make the higher dependency on mycorrhizal associations [48] resulting in an extensive growth of fungal hyphae which could increase soil aggregates formation. 

**Figure 2:**
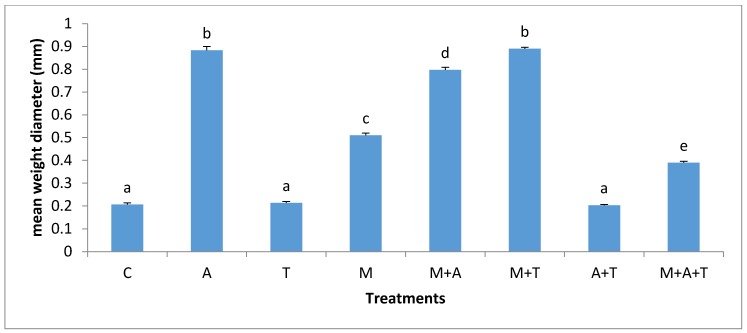
Effect of treatments control (C), algae (A), tea wastes (T), mycorrhizal fungi (M), mycorrhizal fungi + algae (M + A), mycorrhizal fungi + tea wastes (M + T), algae + tea wastes (A + T), and mycorrhizal fungi + algae + tea wastes (M + A + T) on the mean weight diameter (mm) in the saline soil. Bars represent standard errors. Different superscript letters represent the statistical difference (*p* < 0.05).

In the saline soil, the effect of AM fungi on MWD seems to be varied based on the type of wastes (Figure 2). The effect of AM fungi on MWD (*p* < 0.01) increased to a greater extent when M combined with T and to a lesser extent when added alone. The components of tea and algae such as amino acids, polyphenol acid, and carbohydrates might have adjusted soil pH, leading to an increase in microbial community and organic carbon which considered one of the main binding agents of soil aggregates [47] causing a higher MWD. Moreover, the new enhanced conditions of the soil gave a good chance to AM fungi to grow perfectly in the saline soil, encouraging the entanglements of soil particles which can result to a developed soil structure. It has appeared that the addition of AM fungi without organic wastes in the saline soil did not increase MWD to a certain level as other treatments did; this status happened because of the higher concentrations of sodium *(*Na*^+^ )* and chloride *(*Cl*^−^)* ions in the saline soil in which toxic ions might limit AM fungi. This can suggest the importance of the combination of tea wastes with AM fungi to increase soil aggregate formation. 

### 3.3. Effect of Treatments on Bacterial and Fungal Community

**Figure 3:**
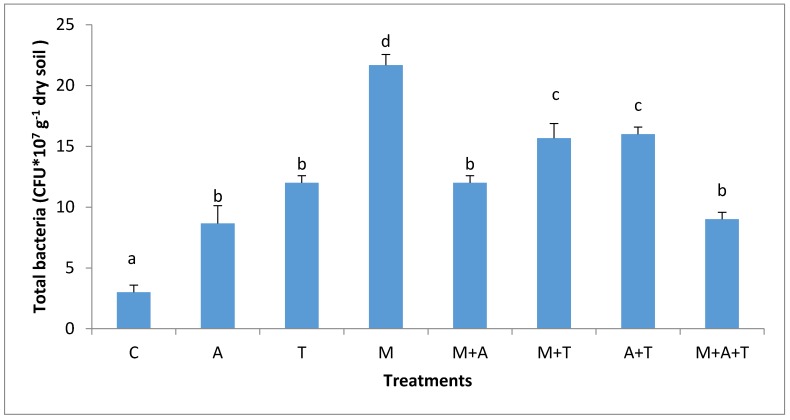
Effect of treatments control (C), algae (A), tea wastes (T), mycorrhizal fungi (M), mycorrhizal fungi + algae (M + A), mycorrhizal fungi + tea wastes (M + T), algae + tea wastes (A + T), and mycorrhizal fungi + algae + tea wastes (M + A + T) on bacterial population (CFU × 10^6^ g^−1^ dry soil) in the non-saline soil. Bars represent standard errors. Different superscript letters represent the statistical difference (*p* < 0.05).

**Figure 4:**
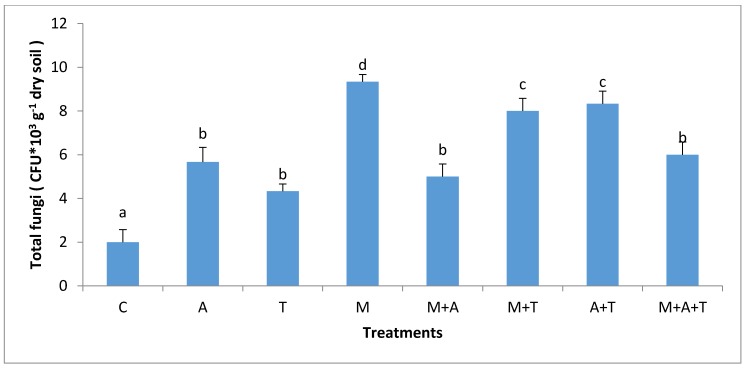
Effect of treatments control (C), algae (A), tea wastes (T), mycorrhizal fungi (M), mycorrhizal fungi + algae (M + A), mycorrhizal fungi + tea wastes (M + T), algae + tea wastes (A + T), and mycorrhizal fungi + algae + tea wastes (M + A + T) on fungal population (CFU × 10^3^ g^−1^ dry soil) in the non-saline soil. Bars represent standard errors. Different superscript letters represent the statistical difference (*p* < 0.05).

The effect of AM fungi on bacterial and fungal communities was different based on the type of organic wastes and soil salinity level. In non-saline soil, the results showed that the highest significant increase of the bacterial and fungal communities (*p* < 0.01; *p* < 0.01) was when M added without organic wastes compared to the rest of the treatments (Figure 3 and Figure 4). AM fungi play an important role in stimulating microbial community via fungal products such as glomalin and glycoprotein [49], these products are a vital source of energy for bacterial and fungal communities. Furthermore, AM fungi can increase the readily available carbon which promotes the growth of bacteria and fungi. Additionally, the products of AM fungi contain low-molecular-weight sugars and organic acids might provoke the bacterial and fungal population in the soil. These findings were consistent with those of a previous paper [22] who emphasized that AM fungi had an important role in increasing the bacterial community.

It was noteworthy that the combination of AM fungi with tea wastes was more effective in increasing the bacterial population (*p* < 0.01) than the single addition of the tea wastes treatment. Tea wastes have small C:N ratio may have been rapidly decomposed by microbes, supporting microbial community for a short period, whereas when AM fungi mixed with the tea wastes, the exudates of AM fungi and a presence of protein and amino acids from the tea wastes might become a major stimulator for the bacterial growth. The authors of a previous paper [50] found that the addition of tea residues improved the biological properties of soil.

**Figure 5:**
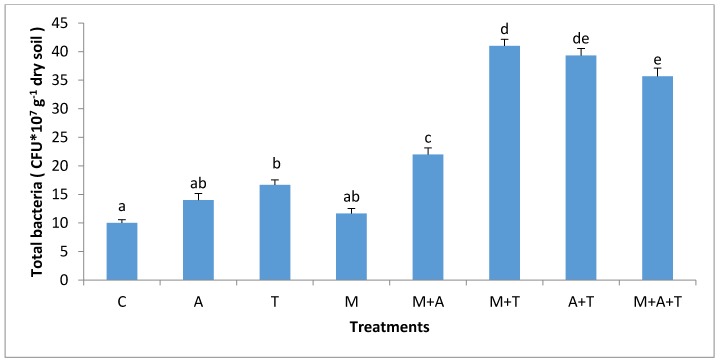
Effect of treatments control (C), algae (A), tea wastes (T), mycorrhizal fungi (M), mycorrhizal fungi + algae (M + A), mycorrhizal fungi + tea wastes (M + T), algae + tea wastes (A + T), and mycorrhizal fungi + algae + tea wastes (M + A + T) on bacterial population (CFU × 10^6^ g^−1^ dry soil) in the saline soil. Bars represent standard errors. Different superscript letters represent the statistical difference (*p* < 0.05).

**Figure 6:**
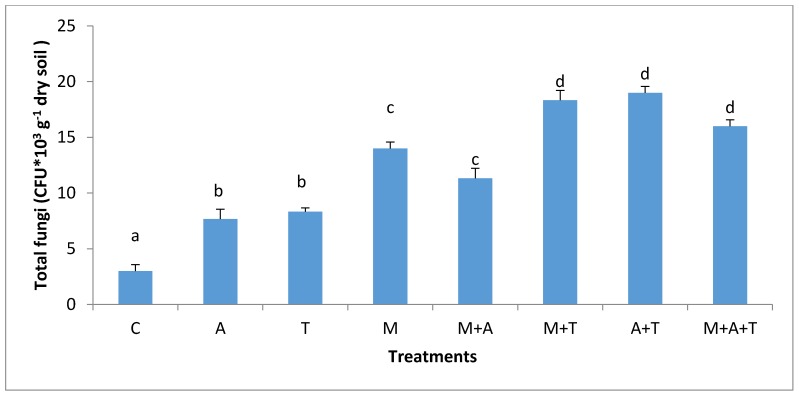
Effect of treatments control (C), algae (A), tea wastes (T), mycorrhizal fungi (M), mycorrhizal fungi + algae (M + A), mycorrhizal fungi + tea wastes (M + T), algae + tea wastes (A + T), and mycorrhizal fungi + algae + tea wastes (M + A + T) on fungal population (CFU × 10^3^ g^−1^ dry soil) in the saline soil. Bars represent standard errors. Different superscript letters represent the statistical difference (*p* < 0.05).

In the saline soil, the scenario was totally different from the non-saline soils; the highest significant increase in bacterial and fungal communities (*p* < 0.01) was in M + T treatment compared to the rest of the treatments (Figure 5 and Figure 6), although it was not significantly different from A + T treatment. The main reason for this phenomenon is that the tea wastes contain organic acids and polyphenols might participate in alternating the high pH of the saline soil, encouraging AM fungi to grow up intensively leading to an increase in mycorrhizal fungal products which can favorably increase the bacterial and fungal population in saline soils. Contrary to our expectations, A and M did not increase the bacterial community as compared to the control treatment. This suggests that A and M additions alone in the saline soil were unable to increase the bacterial population. The possible interpretation is that firstly, the C:N ratio of algal dried biomass is (20:1) which might have degraded rapidly leading to a reduced bacterial community. Secondly, the quantities of algal acids and mycorrhizal exudates seem to be low and have no chance to modify the high pH of the saline soil leading to a diminished aid for the bacterial population.

### 3.4. Effect of Treatments on Soil Organic Carbon 

**Figure 7:**
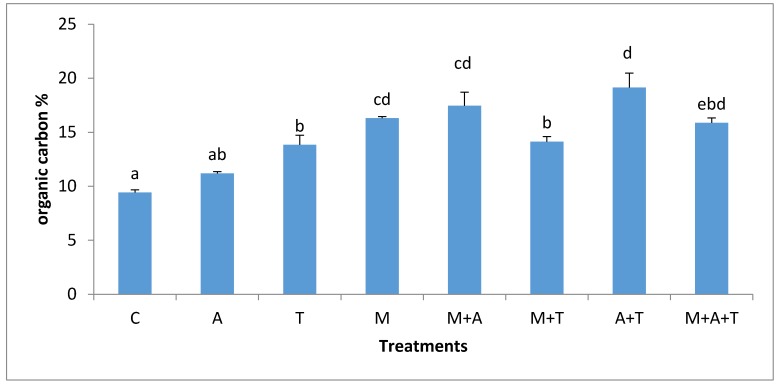
Effect of treatments control (C), algae (A), tea wastes (T), mycorrhizal fungi (M), mycorrhizal fungi + algae (M + A), mycorrhizal fungi + tea wastes (M + T), algae + tea wastes (A + T), and mycorrhizal fungi + algae + tea wastes (M + A + T) on organic carbon (%) in the non-saline soil. Bars represent standard errors. Different superscript letters represent the statistical difference (*p* < 0.05).

In the non- saline soil, the highest percentage of organic carbon (*p* < 0.01) was in A + T treatment (Figure 7) but it was not significantly different from M, M + A and M + A + T treatments. A did not improve the percentage of organic carbon significantly compared to control, but when A combined with AM fungi, organic carbon raised significantly. These results reflected the importance of the combination of AM fungi with algae in enhancing the percentage of organic carbon in the soil. AM fungi can support soil organic carbon through the release of glomalin and chitin [51,52], which might undoubtedly increase soil organic carbon content. More importantly, AM fungi can contribute to the carbon sequestration by increasing the soil aggregates formation in which more carbon can be protected for longer periods. 

**Figure 8:**
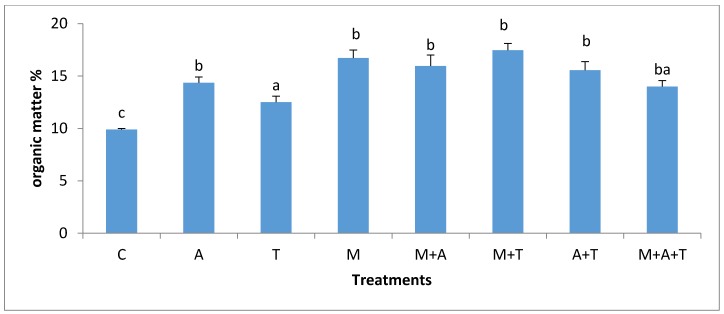
Effect of treatments control (C), algae (A), tea wastes (T), mycorrhizal fungi (M), mycorrhizal fungi + algae (M + A), mycorrhizal fungi + tea wastes (M + T), algae + tea wastes (A + T), and mycorrhizal fungi + algae + tea wastes (M + A + T) on organic carbon (%) in the saline soil. Bars represent standard errors. Different superscript letters represent the statistical difference (*p* < 0.05).

In saline soils, it was observed (Figure 8) that the highest significant increase in the percentage of organic carbon was when M combined with T, although this was not significantly different from M and M + A treatments. It was also found that the T alone did not improve organic carbon to a greater extent. However, when T mixed with M, the results determined a significant increase in the percentage of organic carbon. Polyphenols and amino acids, as well as carbohydrates in tea wastes can create good environmental conditions for the AM fungi to produce more glomalin, which could participate in the recovery of the amount of organic carbon in the soil. The organic carbon content in M + T treatment behaved differently in both soils. The possible explanations can be firstly attributed to the bacterial populations which were substantially higher in M + T treatment of the saline soil (Figure 5); these can have a beneficial support to the soil organic carbon percentage. Authors of a previous paper [47] mentioned that the microbial decomposition of litter can increase the fats, mucilages, waxes, humic compounds, and protein in soil leading to a definite improvement in soil organic carbon content. Secondly, it was noted a high MWD (*p* < 0.01) in M + T treatment in saline soil; these can be undeniable evidence supporting the protection of organic carbon in the soil. Thirdly and more importantly, the corn yield in the saline soil was highly improved by M + T treatment than the non-saline soil. The presence of roots exudates might contribute to organic carbon enhancements [14]. Thus, it is clear that under the stressed saline soil, AM fungi were well associated with the corn plant (Figure 11) to obtain its benefits to overcome the salinity effect. Whilst, under non-saline soil, the corn plant might not need an urgent association with AM fungi because of the teas wastes might have enriched the need of plant of nutrients resulting to lower fungal and roots exudates. The good associations between plant and AM fungi could increase the soil organic carbon content. 

### 3.5. Effect of Treatments on the Infection Rate and Spores Density 

**Figure 9:**
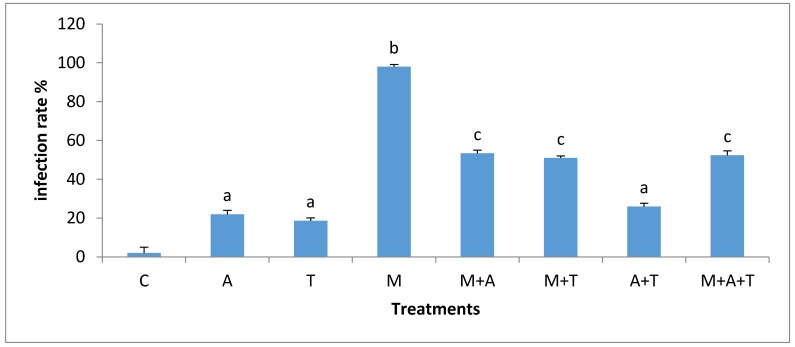
Effect of treatments control (C), algae (A), tea wastes (T), mycorrhizal fungi (M), mycorrhizal fungi + algae (M + A), mycorrhizal fungi + tea wastes (M + T), algae + tea wastes (A + T), and mycorrhizal fungi + algae + tea wastes (M + A + T) on infection rate (%) in the non-saline soil. Bars represent standard errors. Different superscript letters represent the statistical difference (*p* < 0.05).

**Figure 10:**
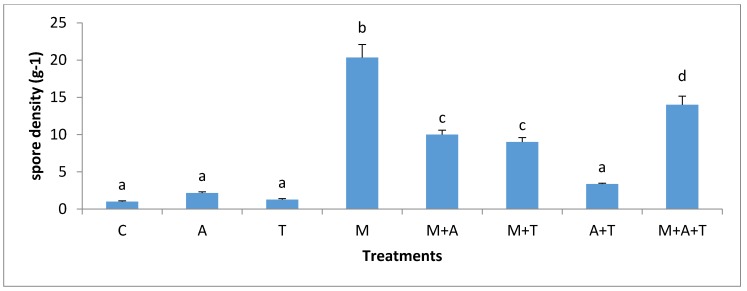
Effect of treatments control (C), algae (A), tea wastes (T), mycorrhizal fungi (M), mycorrhizal fungi + algae (M + A), mycorrhizal fungi + tea wastes (M + T), algae + tea wastes (A + T), and mycorrhizal fungi + algae + tea wastes (M + A + T) on spore density (g^−1^) in the non-saline soil. Bars represent standard errors. Different superscript letters represent the statistical difference (*p* < 0.05).

The effect of treatments on the mycorrhizal infection rate and spore density varied significantly according to the nature of substances and soil salinity level. In non-saline soil, the highest increases of mycorrhizal infection rate (*p* < 0.01) and spores density (*p* < 0.01) were in M treatment compared to the rest of the treatments (Figure 9 and Figure 10). It is probable that the improvement in soil structure (Figure 1) and microbial population (Figure 3 and Figure 4) when AM fungi were added have a direct effect on increasing mycorrhizal infection rate and spores density. Healthy soil has decent amounts of microbial mucilages, carbon, oxygen, and water allowing AM fungi to extend their fungal hyphae in the soil to meet plant roots resulting to an intensive mycorrhizal infection rate. In these regards, the penetration of roots can easily be monitored due to a higher MWD (Figure 1) which lead to a reduction in soil bulk density and soil compaction; thus the mycorrhizal infection rates were highly presented. In addition, this study showed a significant improvement in mycorrhizal infection rates and the spores density when M mixed with T or A in a comparison to the single additions of T and A treatments. These circumstances are the result of the improvements in soil structure, which facilitated the movement of roots and fungal hyphae after the combination of AM fungi with A and T. Moreover, it was recorded an increase in total bacteria and fungi after the combination of AM fungi with A and T, which in turn could have degraded the organic waste and released their metabolic products to the soil solution so that they can boost roots intensity and AM fungi in soil. 

**Figure 11:**
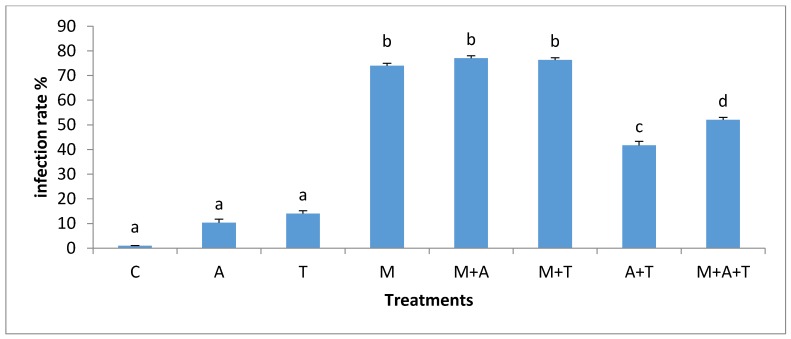
Effect of treatments control (C), algae (A), tea wastes (T), mycorrhizal fungi (M), mycorrhizal fungi + algae (M + A), mycorrhizal fungi + tea wastes (M + T), algae + tea wastes (A + T), and mycorrhizal fungi + algae + tea wastes (M + A + T) on infection rate (%) in the saline soil. Bars represent standard errors. Different superscript letters represent the statistical difference (*p* < 0.05).

**Figure 12:**
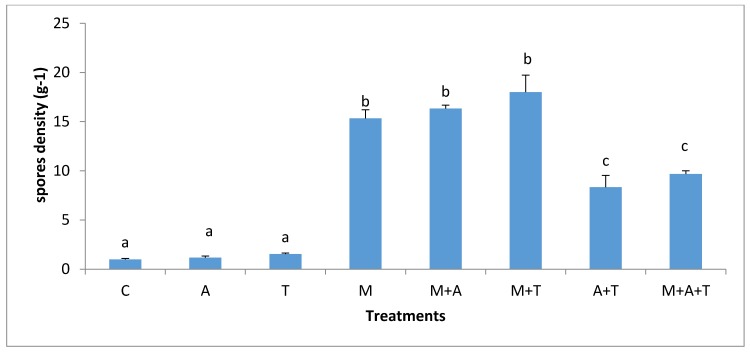
Effect of treatments control (C), algae (A), tea wastes (T), mycorrhizal fungi (M), mycorrhizal fungi + algae (M + A), mycorrhizal fungi + tea wastes (M + T), algae + tea wastes (A + T), and mycorrhizal fungi + algae + tea wastes (M + A + T) on spore density (g^−1^) in the saline soil. Bars represent standard errors. Different superscript letters represent the statistical difference (*p* < 0.05).

In the saline soils, it was recorded significant increases of mycorrhizal infection rates (*p* < 0.01) and spores density (*p* < 0.01) in M + T, M, and M + A treatment compared to A + T, T, and A treatments (Figure 11 and Figure 12). To interpret these results, the properties of organic wastes are critical for increasing mycorrhizal infection rates and spore density in soils. For instances, the combination of AM fungi with tea wastes or algal biomass was more evident in increasing mycorrhizal infection rate in saline soil than non-saline soil. The tea wastes and algae have sufficient amounts of polyphenols and organic acids which could contribute to the modification of high pH, these conditions can subsequently give soil microbes a greater chance to release microbial mucilages, which aid fungal hyphae and roots network in the soil, providing a magnificent opportunity to AM fungi to increase the rate of infection. Researchers have shown that the AM fungi under saline stress conditions absorbed higher amounts of nutrients, thus producing a large density of roots and yield, as well as an increase in the infection rate [4,6].

It is worth mentioning, and based on our data, that the combination of AM fungi with A or T increased the organic carbon content and improved the soil structure causing an enhanced water and aerobic relations, resulting in a higher penetration of the roots in the soil, which encouraged the chances of fungal hyphae to face plant roots to increase the infection rate. It is clear that the availability of organic materials for AM fungi under saline stressed conditions might increase the adaption of AM fungi to overcome the stressed conditions resulting in a higher infection rate. Some studies have identified the vital role of the combination of organic matter with AM fungi in increasing the mycorrhizal infection rate and its impact on the plant growth [53]. Furthermore, under high saline stress conditions, mycorrhizal hyphae might begin to absorb nutrients, water to increase the infection rate in order to overcome the stressed conditions [12,54].

### 3.6. Effect of Treatments on Corn Plant Yield 

**Figure 13:**
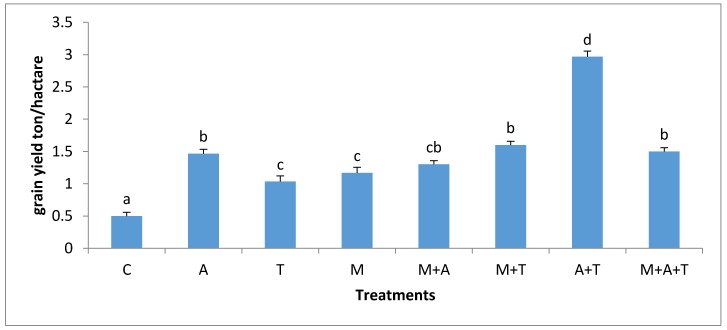
Effect of treatments control (C), algae (A), tea wastes (T), mycorrhizal fungi (M), mycorrhizal fungi + algae (M + A), mycorrhizal fungi + tea wastes (M + T), algae + tea wastes (A + T), and mycorrhizal fungi + algae + tea wastes (M + A + T) on grains yield (ton/hectare) in the non-saline soil. Bars represent standard errors. Different superscript letters represent the statistical difference (*p* < 0.05).

The grains yield of corn plant influenced markedly by the combined treatments. In the non-saline soil, the highest significant grains yield of the corn plant (*p* < 0.01) was when T combined with A compared to all the treatments (Figure 13). The presence of polyphenols acids, protein, nutrients, carbohydrates, and amino acids in these materials might have encouraged corn plant to produce more grains yield. Moreover, there was a sufficient amount of organic carbon in this treatment (Figure 7) which might have aided grains yield production. Organic carbon plays a key role in improving growth of the plant, soil structure and soil microorganisms [44,55]. The accumulated organic carbon can increase the density of roots, which are capable of absorbing nutrients leading to an improved grain yield. This study is consistent with that of a previous paper [56] whose authors concluded that algae are an important biomass that improves the total yield of maize. This study also in accordance with that of a previous paper [57] whose authors found that tea wastes increased the productivity of maize and improved soil microbial activity. The incorporation of M with T significantly improved grains yield of maize plant compared to the single addition of T or M treatments. Tea wastes are a natural organic fertilizer containing substantial amounts of nutrients and organic acids, which contribute to increase the fertility value of soil facilitating the task of AM fungi to uptake more phosphorus, potassium and nitrogen causing a subsequent increase in the grains yield. In addition, AM fungi can produce growth regulators such as auxins, gibberellins, cytokines, amino acids, and vitamins which might enhance the plant root and grains yield [58]. More importantly, the inoculation of AM fungi alone did not increase the grains yield of maize plant as compared to A + T treatment. This may be due to the presence of a large number of bacteria and fungi in M treatment (Figure 3 and Figure 4) which might have competed with the AM fungi for the necessary plant nutrients leading to a suppressed development of yield grains in the non-saline soil.

**Figure 14:**
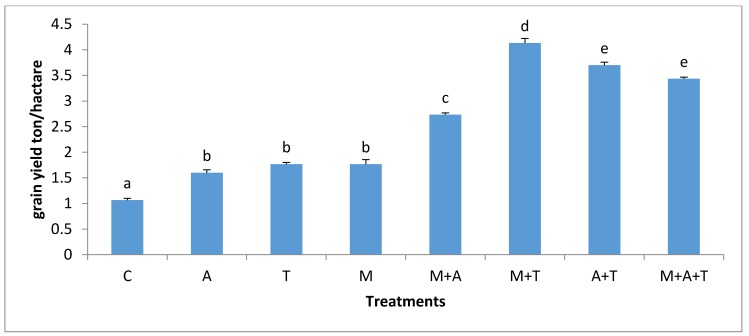
Effect of treatments control (C), algae (A), tea wastes (T), mycorrhizal fungi (M), mycorrhizal fungi + algae (M + A), mycorrhizal fungi + tea wastes (M + T), algae + tea wastes (A + T), and mycorrhizal fungi + algae + tea wastes (M + A + T) on grains yield (ton/hectare) in the saline soil. Bars represent standard errors. Different superscript letters represent the statistical difference (*p* < 0.05).

In the saline soils, it was noted a higher significant increase of the total grains yield of the maize plant (*p* < 0.01) in M + T treatment (Figure 14). This was due to the role of tea wastes which have believed to be a soil conditioner because of their contents of organic acids, protein, and polyphenols which might have modified the high pH of the saline soil. In these certain events, soil microbial activity could be ameliorated by the new habitat leading to more decomposable materials in which more unavailable nutrients converting to available forms causing an improved plant growth. Furthermore, this study has enormous evidences confirmed that the incorporation of AM fungi with tea wastes increased the adaption of the corn plant to salinity through enhancing the mean weight diameter, organic carbon content, and bacterial and fungal abundance. An improvement in soil structure can enhance the soil moisture content and water infiltration leading to perfect roots density and implications for the nutrients uptake and productivity [59]. Moreover, organic carbon can increase the plant growth by enhancing the biophysical properties of soil [60,61,62,63,64]. Furthermore, fungal hyphae were reported to increase glomalin which is one of the most important exudates of fungi causing an increase in the microbial activity and organic carbon in soil [52] contributing to increases in the maize grains yields.

More importantly, tea wastes have large quantities of carbohydrates, polyphenols, and zinc (Table 2), which can promote the plant growth. Past studies have shown that AM fungi can consolidate the plant adaption to salinity by improving nutrients uptake and limiting the absorption of sodium and chloride ions by reducing their movements to the aerial parts of the plant [2,6] and improving water uptake by hyphae [65]. Concurrently, AM fungi improved the physiological status of the plant by regulating the osmotic pressure and accumulating of carbohydrates in the saline soils as a direct means to overcome salinity problems [66,67].

## 4. Conclusions

A current conclusion of this experiment is that the incorporation of AM fungi with tea wastes in the saline soil is considered the best homogeneous combination to improve soil biological properties and grains yield of the corn plant. In general, the application of AM fungi, tea wastes, and algal dried biomass increased microbial activity compared to the control, while, the incorporation of AM fungi with tea wastes or algal dried biomass reduced CO_2_ release compared to the single addition of tea wastes or algal dried biomass treatments. Therefore, AM fungi may play a vital role in the carbon sequestration. In addition, this study showed that the inoculation of AM fungi in the non-saline soil increased significantly mean weight diameter, total bacteria, total fungi, and infection rate as well as spores density, whereas the opposite was true in the saline soil, AM fungi showed a limited capability of developing soil biological properties because of the salinity impact. In conclusion, the incorporation of AM fungi with tea wastes might have promoted the adaption of the corn plant to salinity conditions by enhancing mean weight diameter, organic carbon content, bacterial and fungal abundance, infection rate, and spores density.

## Figures and Tables

**Table 1:** Some physical and chemical characteristics of soils.

Properties	Units	Saline Soil	Non-Saline Soil
EC	(dS/m)	7.9	3.6
pH		7.76	7.83
P	ppm	2.26	1.81
N	ppm	0.071	0.057
K	ppm	35.2	94.8
CaCo_3_	%	181.24	202.87
CaSO_4_	g/kg^−1^	8.24	2.21
**Texture**		**Sandy Clay**	**Clay Loam**
clay	g/kg^−1^	46	34
silt	g/kg^−1^	7	37
sand	g/kg^−1^	47	29
Ca	Mg/L	19	29
Mg	Mg/L	26	111
Cl^−^	Mg/L	78.96	25.38
CO_3_^−2^	Mg/L		
HCO^−3^	Mg/L	4.2	5
O.M	g/kg^−1^	1.41	1.13
Bulk density	g/cm^3^	1.61	1.29

**Table 2:** Chemical composition of the tea wastes and algal dried biomass.

Properties	Tea Waste	Algae
carbon	30	28
carbohydrates %	1.3	0.85
Zn ppm	25	18
Fe ppm	33	37
Total polyphenols %	20	0.7
Amino acids %	2.0	18
Protein %	20	44
N %	1.5	1.6
p %	0.021	0.331
K %	1.1	1.45
C/N	19.3	20.8

**Table 3:** Effect of treatments control (C), algae (A), tea wastes (T), mycorrhizal fungi (M), mycorrhizal fungi + algae (M + A), mycorrhizal fungi + tea wastes (M + T), algae + tea wastes (A + T) and mycorrhizal fungi + algae + tea wastes (M + A + T), and incubation period (2 day, 4 day, 8 day, 12 days, and 30 days) on the microbial activity (CO_2_-C Mg g^−1^) in the non-saline soil. Mean ± standard error: means with a common letter superscript do not differ significantly (*p* < 0.05).

Treatments	Incubation Periods					
	2 Days	4 Days	8 Days	12 Days	30 Days	Mean
C	14.00 ± 0.57 ^b^	13.00 ± 0.57 ^b^	12.00 ± 0.57 ^b^	9.00 ± 0.57 ^b^	07.00 ± 0.57 ^b^	11.0 ± 0.73 ^b^
A	21.00 ± 0.57 ^a^	19.05 ± 0.53 ^a^	17.90 ± 0.58 ^a^	16.00 ± 0.57 ^a^	14.00 ± 0.57 ^a^	17.5 ± 0.68 ^a^
T	23.00 ± 0.57 ^d^	19.33 ± 0.88 ^d^	18.42 ± 0.81 ^ac^	16.17 ± 0.43 ^a^	14.66 ± 0.66 ^a^	18.3 ± 0.80 ^c^
M	18.46 ± 0.06 ^c^	18.00 ± 0.03 ^ca^	15.43 ± 0.40 ^a^	14.55 ± 0.51 ^a^	12.17 ± 0.61 ^a^	15.7 ± 0.63 ^d^
M + A	18.25 ± 0.06 ^c^	17.96 ± 0.18 ^c^	16.57 ± 0.47 ^a^	15.47 ± 0.03 ^a^	11.03 ± 0.24 ^c^	15.8 ± 0.70 ^d^
M + T	17.16 ± 0.06 ^c^	17.52 ± 0.03 ^c^	13.62 ± 0.47 ^ab^	13.62 ± 0.95 ^a^	11.51 ± 0.03 ^c^	15.3 ± 0.65 ^d^
A + T	19.38 ± 0.30 ^ca^	18.70 ± 0.51 ^ca^	19.66 ± 0.88 ^c^	15.83 ± 0.44 ^a^	13.50 ± 0.28 ^a^	17.4 ± 0.66 ^a^
M + A + T	19.03 ± 0.06 ^ca^	17.74 ± 0.16 ^c^	15.91 ± 0.37 ^a^	14.66 ± 0.54 ^a^	11.07 ± 0.67 ^c^	15.6 ± 0.75 ^d^
mean	18.78 ± 0.52 ^e^	17.66 ± 0.41 ^e^	16.57 ± 0.48 ^f^	14.41 ± 0.48 ^g^	11.87 ± 0.49 ^h^	

**Table 4:** Effect of treatments control (C), algae (A), tea wastes (T), mycorrhizal fungi (M), mycorrhizal fungi + algae (M + A), mycorrhizal fungi + tea wastes (M + T), algae + tea wastes (A + T) and mycorrhizal fungi + algae + tea wastes (M + A + T), and incubation period (2 day, 4 day, 8 day, 12 days, and 30 days) on the microbial activity (CO_2_-C Mg g^−1^) in the saline soil. Mean ± standard error: means with a common letter superscript do not differ significantly (*p* < 0.05).

Treatments	Incubation Periods					
	2 Days	4 Days	8 Days	12 Days	30 Days	Means
C	13.33 ± 0.88 ^a^	13.00 ± 0.57 ^a^	13.66 ± 0.33 ^a^	12.00 ± 0.57 ^a^	06.00 ± 0.57 ^a^	11.60 ± 0.79 ^a^
A	21.66 ± 0.88 ^bd^	21.66 ± 0.88 ^d^	17.50 ± 0.28 ^d^	15.83 ± 0.16 ^b^	13.29 ± 0.59 ^b^	17.99 ± 0.91 ^b^
T	23.00 ± 0.57 ^d^	24.00 ± 0.57 ^d^	18.01 ± 0.32 ^d^	17.15 ± 0.51 ^d^	14.40 ± 0.35 ^d^	19.31 ± 0.98 ^c^
M	18.25 ± 0.57 ^c^	18.00 ± 0.35 ^c^	16.85 ± 0.90 ^c^	14.65 ± 0.16 ^c^	11.77 ± 0.16 ^cb^	15.90 ± 0.67 ^ed^
M + A	17.71 ± 0.73 ^c^	17.32 ± 0.47 ^c^	15.19 ± 0.69 ^ca^	14.92 ± 0.48 ^c^	11.77 ± 1.25 ^c^	14.96 ± 0.82 ^e^
M + T	19.35 ± 0.06 ^c^	17.25 ± 0.28 ^c^	17.38 ± 0.35 ^c^	14.74 ± 0.06 ^c^	09.86 ± 1.20 ^c^	15.71 ± 0.90 ^ed^
A + T	19.50 ± 0.28 ^cb^	18.93 ± 0.52 ^c^	18.00 ± 0.28 ^c^	15.06 ± 0.52 ^c^	12.29 ± 0.35 ^cb^	16.75 ± 0.73 ^d^
M + A + T	18.92 ± 0.06 ^c^	17.65 ± 0.17 ^c^	16.75 ± 0.19 ^c^	14.19 ± 0.80 ^c^	10.60 ± 0.80 ^cb^	15.62 ± 0.79 ^e^
mean	18.96 ± 0.58 ^a^	18.48 ± 0.74 ^a^	16.67 ± 0.33 ^b^	14.81 ± 0.32 ^c^	10.98 ± 0.52 ^d^	
